# Fatigue Resistance of Cast-on Implant Abutment Fabricated with Three Different Alloys

**DOI:** 10.1055/s-0041-1742124

**Published:** 2022-02-23

**Authors:** Usanee Puengpaiboon, Pavinee Padipatvuthikul Didron

**Affiliations:** 1 Department of General Dentistry, Faculty of Dentistry, Srinakharinwirot University, Bangkok, Thailand

**Keywords:** dental implant, cast-on abutment, fatigue resistance, mechanical failure

## Abstract

**Objectives** This study aimed to evaluate fatigue resistance of cast-on implant abutment using three alloys.

**Materials and Methods** Forty specimens of implant-supported crowns were prepared; Group 1 (TA) stock titanium abutments, Group 2 (GS) abutment cast with 40% gold alloy, Group 3 (GP) abutment cast with palladium alloy, and Group 4 (CN) abutment cast with nickel–chromium alloy. Specimens were cyclic loaded at 20 Hz, starting from 200 N (5,000 cycles), followed by stepwise loading of 400, 600, 800, 1,000, 1,200, 1,400, 1,600, and 1,800 N (30,000 cycles/step). Specimens were loaded until failure or reached 245,000 cycles.

**Statistical Analysis** The withstand cycles were analyzed using one-way analysis of variance and Weibull survival analysis. Fracture surfaces were examined using scanning electron microscopy.

**Results** The results of withstand cycles were TA (189,883 ± 22,734), GS (195,028 ± 22,371), GP (187,662 ± 22,555), and CN (200,350 ± 30,851). The statistical analysis showed no significant difference between the groups ( *p* = 0.673).

**Conclusion** Although CN has higher Weibull characteristic strength which means greater durability, its lower Weibull modulus demonstrated less structural reliability. Consistent failures at implant fixture level were also found in CN group.

## Introduction

Gold alloy has been accepted as the gold standard material for fabrication of dental implant casting custom abutment. 1 2 Gold price has continuously increased over the past decades, and at the same time, alternative alloys such as nickel–chromium and palladium alloys have been developed. Nickel–chromium is one of the most commonly used alloys in prosthetic dentistry. This alloy is considerably much cheaper than gold alloy and offers good biocompatibility, corrosion resistance, and castability. 1 3 However, thick oxide layer from nickel–chromium casting procedure can cause surface roughness that is difficult to smooth-up due to its high surface hardness, this results in misfit of abutment-implant connection. 3 4 Palladium alloy offers good mechanical properties including flexural strength, stiffness, and durability. It also poses excellent tarnish/corrosion resistance and good biocompatibility in the oral environment. 5 For alternative cast-on abutment, there are many implant systems in the market in which those cast-on abutment can be fabricated using nonprecious alloy to reduce the overall cost. In a study by Kano et al, 6 the cast cobalt–chromium abutments showed greater rotational misfit compared with stock titanium abutment. Similar conclusions were drawn by Barbosa et al, 1 who reported that one-piece cast frameworks made with cobalt–chromium alloy had the worst result for passive fit compared with commercially pure titanium and nickel–chromium–titanium alloys, but their vertical fit was comparable.

Despite long-term reliability of dental implant with survival rate of 95% after 5 years and 90% after 15 years, 7 8 both biological and mechanical complications still occur. 7 9 Fatigue is one of the factors that causes mechanical failure. Mechanical fatigue test with a cyclic loading is a laboratory method used for evaluating clinical reliability of dental implants. 10 This test aims to simulate the clinical intraoral conditions and masticatory function. 11 Fatigue tests have been used in many of dental implant studies, but the majority had performed in stock titanium or zirconia abutment. 12 13 14

Cast-on abutment for screw-retained prosthesis has the advantage of predictable retrievability. 15 16 It can be used in patients with minimal interocclusal space of less than 4 mm or when the implant orientation is misaligned more than 30 degrees. 15 17 In patients with limited interocclusal space at edentulous area and economically compromised, using nonprecious cast-on abutment could be a good solution. However, there is still insufficient data regarding fatigue resistance of cast-on implant abutments. The purpose of this study was, therefore, to evaluate and compare fatigue resistance of cast-on abutments, cast with three different alloys as follows: gold, palladium, and nickel–chromium alloys. Apart from the difference in mechanical properties of the chosen alloys, these three types of alloy also represent different level of material cost. The hypothesis of this study is that “the type of alloy used for cast-on abutments does not affect fatigue resistance of the implant.”

## Materials and Methods

### Specimen Preparation

**Fig. 1:**
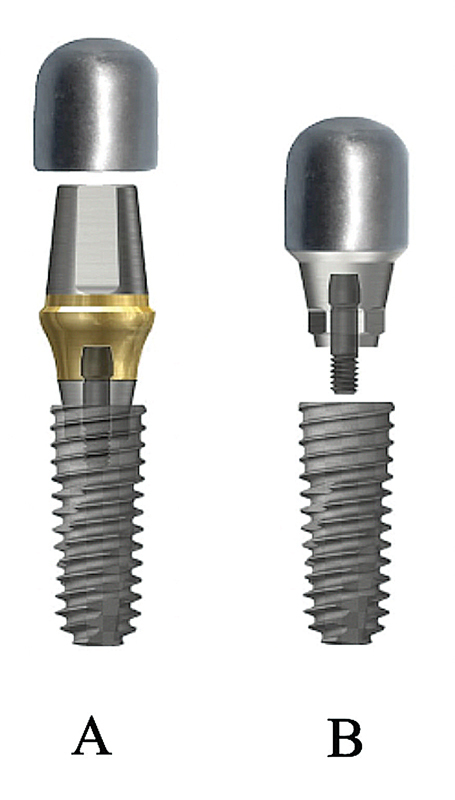
( **A** ) Implant components in Group TA. ( **B** ) Implant components in Groups GS, GP, and CN.

Forty specimens of fixture, with internal connection (Ø 4.5 mm, length 10 mm, TSIII; Osstem, Seoul, Korea, Lot No. FTP5A348) and abutment, representing single implant-supported crown ( Fig. 1 ) were prepared and divided into four groups ( *n* = 10) including Group TA (control): stock titanium abutment, Group GS: gold cast abutment cast with semiprecious alloy, Group GP: gold cast abutment with palladium alloy, and Group CN: nonprecious cast abutment cast with nickel–chromium alloy. All materials and instruments listed by the manufacturer and composition of the abutments are shown in Table 1 .

**Table 1:** Types of abutments and casting materials

Group	Type of prosthesis	Abutment	Materials										
			Alloys	Composition									Melting interval (°C)
				Au	Pd	Ag	Cu	Ni	Cr	Mo	Sn	Etc.	
TA (control)	Cement-retained crown	Stock titanium abutment (Osstem, Korea; Lot No. PGA15A219)	Titanium alloy grade 5	–	–	–	–	–	–	–	–	–	–
GS	Screw-retained crown	Gold cast abutment (Osstem, Korea; Lot No. PGG15B019)	Semiprecious (Minigold, Ivoclar Vivadent, United States; Lot No. V41451PM)	40	4	47	7.5	–	–	–	–	Zn, In, Ir	865–945
GP	Screw-retained crown	Gold cast abutment (Osstem, Korea; Lot No. PGG15B019)	Pd based (W-1, Ivoclar Vivadent, United States; Lot No. W03211QG)	–	53.3	37.7	13	–	–	–	8.5	In, Ru, Li	1,185–1,270
CN	Screw-retained crown	Nonprecious cast abutment (Osstem, Korea; Lot No. PTA15G220)	Ni-Cr (Argeloy N.P.(V), Argen, United States; Lot No. 10311446)	–	–	–	–	76	14	6	–	Al, Be, Fe, Si, C	1,230–1,290

The implant fixtures were mounted centrally and parallel to an implant-holding device and embedded in self-cured resin (Chockfast orange, Shannon, Ireland) following ISO 14801:2016 specification. The resin was poured below the implant platform 3 ± 0.5 mm to simulate the worst-case situation of crestal bone resorption. Artificial crowns were fabricated from hemisphere wax pattern, with a dimension of 6 mm in diameter and 6 mm in height. 18 The wax patterns were invested with phosphate-bonded investment (Bellavest SH; Bego, Bremen, Germany) and cast with the selected alloy at suitable casting temperature ( Table 1 ). After casting, the oxidized layer was removed by 100 µm aluminum oxide particles (Cobra; Renfert GmbH, Hilzingen, Germany) using 60 psi pressure for full metal crown in Group TA, whereas 50 µm glass beads (Rolloblast; Renfert GmbH, Hilzingen, Germany) were used with 60 psi pressure for Groups GS, GP, and CN and followed by ultrasonic cleansing. No further polishing and finishing were performed.

For Group TA (control), specimens were prepared as cement-retained crown to eliminate any effect on abutment connection from the casting process. The stock titanium abutments were torqued to the fixture (30 Ncm), according to manufacturer's recommendation. Ten nickel–chromium crowns were luted and seated on their stock abutments with zinc phosphate cement (HY-Bond Zinc Phosphate Cement; Shofu Dental Corporation, San Marco, California, United States). Excess cement was then removed with microbrush.

For Groups GS, GP, and CN, screw-retained crowns of the cast-on abutments were torqued to the fixture (30 Ncm), according to manufacturer's recommendation. The screw holes were then filled with resin composite (Filtek Z350 XT; 3M ESPE, St. Paul, Minnesota, United States) then light cured for 40 seconds.

### Fatigue Testing

According to ISO 14801:2016, in Dentistry–Implants–Dynamic loading test for endosseous dental implants, all specimens were positioned at 30 ± 2 degrees to the implant axis. 18 The universal testing machine (ElectroPuls E10000; Instron Corporation, Norwood, Massachusetts, United States) was calibrated following the manufacturer's instructions. Dynamic loading fatigue tests were run in dry conditions at room temperature (25 ± 2°C). Cyclic load was programmed using dedicated software (BlueHill version 2.0; Instron Corporation) at a frequency of 20 Hz, starting with 200 N load for 5,000 cycles (preconditioning phase), followed by 400, 600, 800, 1,000,1,200, 1,400, 1,600, and 1,800 N at a maximum of 30,000 cycles for each step. All specimens were loaded until catastrophic failure occurred, or the specimen displaced at least 2 mm from the axis of the dental implant or 245,000 cycles were reached. 19 20 The displacement was automatically measured and detected by the universal testing machine software.

Catastrophic failure was defined as the fracture of any component of the sample. The modes of failure of all samples were recorded and classified into three types as follows: Type 1: fracture at abutment screw and abutment, Type 2: fracture at abutment screw and fixture, and Type 3: fracture at abutment screw, abutment, and fixture. The fracture surface was then examined with scanning electron microscopy (SEM) (JEOL, 6510LV, Japan).

### Statistical Analysis

Group comparisons were analyzed using one-way analysis of variance (ANOVA) and Tukey's post hoc test (α = 0.05). As a supplement to the ANOVA, Weibull survival analysis was performed. The *p* -values < 0.05 were considered statistically significant.

## Results

**Fig. 2:**
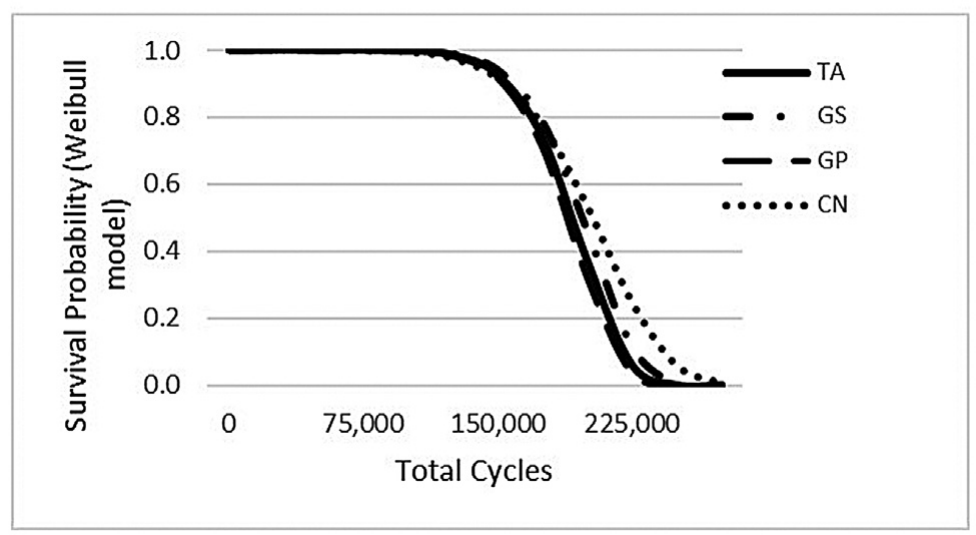
Results of survival probability using Weibull model.

The mean value and standard deviation of total number of cycles are shown in Table 2 . Group CN showed the highest total number of cycles, but no statistically significant differences were found between the groups. Survival probability of Weibull models is shown in Fig. 2 . Group CN has higher Weibull characteristic strength but lower Weibull modulus, which demonstrates greater durability but less structural reliability ( Table 2 ).

**Table 2:** Results of the one-way ANOVA and Weibull test for total number of cycles between various abutments

Group	Mean	SD	Weibull characteristic strength	Weibull modulus
TA	189,883 a	22,734	200,081	8.86
GS	195,028 a	22,371	205,759	8.73
GP	187,662 a	22,555	197,614	8.97
CN	200,350 a	30,851	213,991	6.77

Abbreviations: ANOVA, analysis of variance; SD, standard deviation.

a No significant difference ( *p* -value = 0.673).

**Fig. 3:**
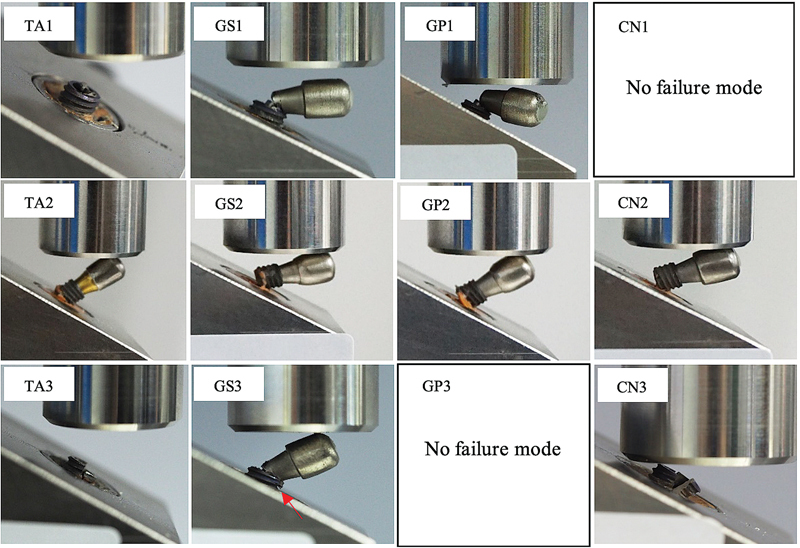
Modes of failure of specimens. Top row: TA1, GS1, and GP1 showed fracture of abutment and abutment screw in Groups TA, GS, and GP, respectively. Middle row: TA2, GS2, GP2, and CN2 showed fracture of fixture and abutment screw in Groups TA, GS, GP, and CN, respectively. Bottom row: TA3, GS3, and CN3 showed fracture of abutment, abutment screw, and fixture in Groups TA, GS, and CN, respectively.

Modes of failures of CN group were all located at the fixtures and abutment screws, whereas in the other groups, their failures predominantly located at abutment and abutment screw level ( Fig. 3 and Table 3 ).

**Table 3:** Modes of failure of specimens

Group	Modes of failure		
	Fractured abutment screw and abutment	Fractured abutment screw and fixture	Fractured abutment screw, abutment, and fixture
TA	6	1	3
GS	7	2	1
GP	9	1	0
CN	0	5	5

**Fig. 4:**
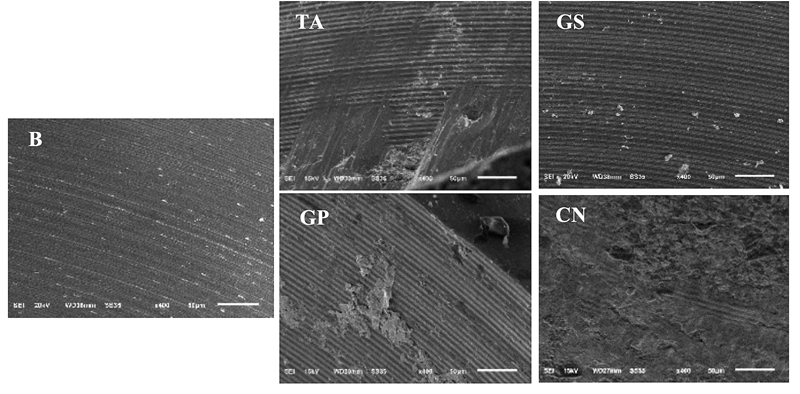
Scanning electron microscopy (×400) of internal implant surface, (B) before fatigue test; (TA) moderate wear in TA group; (GS) minimal wear in GS group; (GP) minimal wear in GP group; (CN) aggressive wear in CN group.

Scanning electron micrographs of internal implant surfaces revealed only minimal damage on implant-abutment connection in GS and GP groups, while the highest wear was observed in CN group ( Fig. 4 ).

**Fig. 5:**
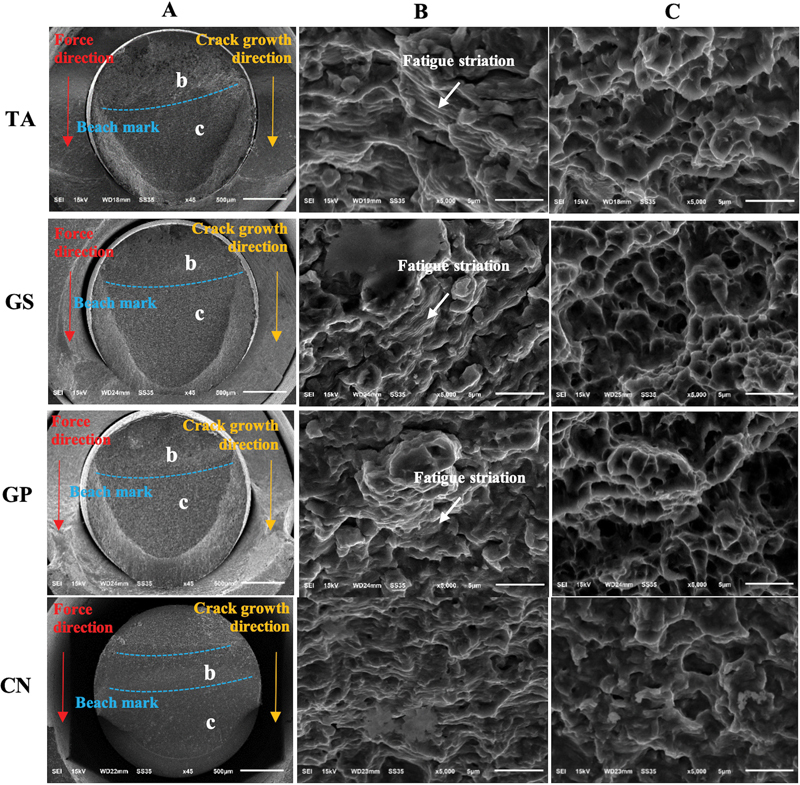
Scanning electron microscopy of abutment screw surfaces, ( **A** ) Force direction and crack growth direction from b (fatigue zone) to c (catastrophic zone). ( **B** ) High-magnification (×5,000) image of fatigue zone. ( **C** ) High-magnification (×5,000) image of catastrophic zone.

Low-magnification SEM micrographs of abutment screw surface show crack growth that originated at the receiving force side. The fatigue crack growth exhibited the boundary of crack progression known as progressive mark or beach mark. Crack progressed until the material had become unstable and overloaded, then catastrophic failure occurred ( Fig. 5A ).

Fig. 5A displays a parallel pattern or fatigue striation, observed in the fatigue zone (b) of high-magnification SEM micrographs of TA, GS, GP groups, which is a character of fatigue failure. However, in CN group, fatigue striations could not be observed because the crack surface was not parallel to SEM image plane ( Fig. 5B ).

High-magnification SEM micrographs of catastrophic zone (c) show characterized microvoids or cup-like depressions or dimples ( Fig. 5C ).

## Discussion

The purpose of this study was to evaluate and compare fatigue resistance of cast-on implant abutment cast with different alloys. Although nonprecious cast abutment group showed the highest total number of cycles, no statistically significant difference was found between the groups. Weibull survival analysis showed that CN group had higher Weibull characteristic strength and lower Weibull modulus which demonstrate greater durability but less structural reliability compared with the other alloys. The primary advantage of nonprecious alloys is their lower cost compare with gold and palladium alloys. At the time of publishing, the cost of nonprecious alloy from Thai dental laboratory is at least 90% lower than gold alloy. For a posterior restoration, the cost difference could be up to 250 USD. In addition, nonprecious alloys demonstrate good mechanical properties, good biocompatibility, and corrosion resistance. 3

A stepwise loading protocol was applied in this study in which a fixed load was applied to determine the total number of cycles, followed by incremental increases in load step up to the upper limit of testing or failure of the specimen. 19 21 This protocol was proved to simulate a clinical situation and shorten the period of testing time. 21 Several studies 19 21 22 23 also had selected the stepwise protocol to accelerate fatigue failure. Regarding the ISO standard 14801:2016 (Dentistry–Implants–Dynamic fatigue test for endosseous dental implants), 18 the test should be at the loading frequency not more than 15 Hz, but the test at low frequency may increase testing time and cost. In a study by Fraga et al, 14 the fatigue strength was not significantly different between the groups using frequency of 2, 10, and 20 Hz; therefore, using frequency of 20 Hz seems to be a good alternative that has been applied in several studies. 19 24

Bonfante and Coelho 25 reported mean maximum bite force of 418 to 690 N in females and 491 to 878 N in males. The range of failure loads in this study were 1,200 to 1,800 N which demonstrated that the specimens in all groups could tolerate a greater load than average occlusal force in natural teeth. The results were similar in several studies. 12 19 20 In our research, the range of the total number of cycles were 180,000 to 230,000 cycles which were higher than previous studies. 19 21 This might be caused by the differences in implant diameter, type of implant abutment connection, type of restorative materials, and the determination of the failure point.

A systematic review 26 reported that 5-year survival rate of screw-retained implant restoration was 96.8%. The technical complications on implant-supported single crown include screw loosening (4.7%), abutment/screw fractures (4.1%), fractures of the veneering material (2.4%), and implant fractures (0.1%). Different failure patterns were observed. Lee et al 27 and Atieh 28 found that the mode of failures often located at implant fixture and screw abutment. On the contrary, Shirazi et al 29 showed the fracture line located only at abutment screw. In this study, the failures were located at fixtures and abutment screws in CN group, but predominantly involved abutments and abutment screws fracture in other groups ( Table 3 ). Such mode of failure can be explained by the higher modulus of elasticity of nonprecious alloys (180–240 GPa) when compared with commercial pure titanium grade 4 (110–150 GPa). The stress distribution then may be concentrated at the implant-abutment complex, especially on the abutment screw. 30 31 Damages of implant-abutment complex can be found at different levels, which need different treatment regimen for repairing. Damage at abutment screw or prosthesis could be easily retrieved or repaired without complication, but the damage at fixture level usually requires a complicated surgery or more extensive treatment. 27 32

The SEM images revealed the highest wear in CN groups. This result is not surprising because the fixtures used in this study made from commercial pure titanium grade 4 which has lower surface hardness and wear resistance than cobalt–chromium cast-on abutment. 30 33

Iwabuchi et al 34 found that both grade 5 titanium alloy and cobalt–chromium alloy have good wear resistance. However, when sliding wear test was performed using aluminum oxide, more surface damage was still found on grade 5 titanium alloy than cobalt–chromium alloy.

Besides mechanical properties of alloy, corrosion resistance and biocompatibility are also important factors to be considered when selecting abutment material. Tuna et al 35 reported that gold alloy and palladium alloy showed high corrosion resistance, in agreement with several studies. 36 37 Palladium alloy, however, may cause allergic reactions in some population. Faurschou et al 38 reported that palladium alloy allergy was found 7.4% in dental patients. While the clinical concern of palladium allergy is still inconclusive, 38 39 40 41 nonprecious alloys have been reported of unstable galvanic corrosion. 42 Lee et al 43 reported the metal ion releasing was increased when nonprecious alloy was in contact with titanium, and it can also cause tissue toxicity around the implant. A study showed that base alloy coupled to CP titanium grade 2 had higher galvanic corrosion than to noble alloy, 44 whereas other studies could not confirm the effect of galvanic corrosion when nonprecious alloy was in contact with titanium. 44 45

Based on fatigue resistance test results in this study, it was found that the use of three types of alloys for cast-on abutment resulted in nonsignificant difference in fatigue resistance. In terms of mechanics, using nonprecious or palladium alloy instead of gold alloy which is the standard for casting custom abutment or screw-retained crown is possible. Regarding the material properties, palladium alloy can be used as a substitute for gold alloy due to the lower melting point than the abutment connection of gold cast abutment.

The SEM images showed worn implant surface where in contact with the cast on connection for the nonprecious abutment group. The worn surface could be a result of micromovement since the thick oxide layer from casting process cannot be eliminated without causing microgap at the implant-abutment connection. 46 In addition, the difference in elastic modulus and hardness may lead to higher wear at the internal fixture surface. 47 48 This must be considered when choosing material because this kind of damage on the implant surface is irreparable.

The fatigue resistance test in this study was tested in accelerated condition which cannot be infer to a comparable fatigue life, but may be assumed as the worst-case scenario that may happen. Therefore, clinically such wear may not occur throughout the dental implant service life. In addition, this study was focused on fatigue resistance of cast-on abutments cast with three different alloys, connected to 4.5 mm diameter implant, without a complete oral environment simulation which could be related to long-term complications. Therefore, further studies and evaluations using different implant diameters, prosthetic designs, and other implant-abutment connection design are needed and will fulfill the knowledge gap.

## Conclusion

Based on the results in this study, no statistically significant difference between the control and experimental groups was found. Although nonprecious cast abutment showed promising fatigue resistance after cyclic loading, the lower Weibull modulus and consistent failures at implant fixture level were also found in this group. These factors should be considered when selecting abutment material.
